# A Robust and Rapid Method of Producing Soluble, Stable, and Functional G-Protein Coupled Receptors

**DOI:** 10.1371/journal.pone.0023036

**Published:** 2011-10-25

**Authors:** Karolina Corin, Philipp Baaske, Deepali B. Ravel, Junyao Song, Emily Brown, Xiaoqiang Wang, Sandra Geissler, Christoph J. Wienken, Moran Jerabek-Willemsen, Stefan Duhr, Dieter Braun, Shuguang Zhang

**Affiliations:** 1 Center for Biomedical Engineering, Massachusetts Institute of Technology, Cambridge, Massachusetts, United States of America; 2 NanoTemper Technologies GmbH, München, Germany; 3 Center for Bioengineering and Biotechnology, China University of Petroleum (East China), Qingdao, Shandong, People's Republic of China; 4 Systems Biophysics, Functional Nanosystems, Ludwig-Maximilians University München, München, Germany; The University of Manchester, United Kingdom

## Abstract

Membrane proteins, particularly G-protein coupled receptors (GPCRs), are notoriously difficult to express. Using commercial *E.coli* cell-free systems with the detergent Brij-35, we could rapidly produce milligram quantities of 13 unique GPCRs. Immunoaffinity purification yielded receptors at >90% purity. Secondary structure analysis using circular dichroism indicated that the purified receptors were properly folded. Microscale thermophoresis, a novel label-free and surface-free detection technique that uses thermal gradients, showed that these receptors bound their ligands. The secondary structure and ligand-binding results from cell-free produced proteins were comparable to those expressed and purified from HEK293 cells. Our study demonstrates that cell-free protein production using commercially available kits and optimal detergents is a robust technology that can be used to produce sufficient GPCRs for biochemical, structural, and functional analyses. This robust and simple method may further stimulate others to study the structure and function of membrane proteins.

## Introduction

G-Protein Coupled Receptors (GPCRs) are the focus of intense research, as they are the largest class of integral membrane proteins and are the targets of ∼50% of pharmaceutical drugs. A critical bottleneck in GPCR studies is the difficulty of expressing soluble and stable receptors in sufficient quantities. A rapid, simple, cost-effective and high-yield method of producing GPCRs is crucial to advance structure and function studies.

Although cell-free *in vitro* translation is a mature technology, it has only recently been used to express and to produce membrane proteins [1]–[16]. This is primarily due to the necessity of including a detergent capable of solubilising and stabilizing the newly synthesized proteins without interfering with transcription or translation. Finding an optimal detergent is expensive and laborious. Several studies indicate that mild detergents like polyoxyethylene derivatives may be effective [3], [14], but only a limited number of proteins have been successfully tested [1]–[15], and published reports have primarily limited their studies to one or few proteins.

Although these results are promising, two areas must still be addressed for cell-free expression to become a practical and widely useful technology for producing GPCRs. First, cell-free expressed proteins must be directly compared to those produced in mammalian cells or purified from native tissues. Many GPCRs require post-translational modifications for structural stability or biological function. *E. coli* and wheat germ extracts do not contain the necessary machinery. Thus, it must be demonstrated that the structure and binding affinities of cell-free expressed receptors are similar to those of native receptors. To the best of our knowledge, none of the published studies has directly compared a cell-free GPCR to a counterpart expressed in mammalian cells to verify that the cell-free constructs are indeed viable. Second, many studies have only used cell-free systems developed in individual laboratories [1]–[13]. In order for cell-free technology to truly benefit the entire GPCR and membrane protein research community, commercial reagents are optimal to minimize variations in sample preparations from each laboratory. It would therefore be advantageous if commercial cell-free systems traditionally used for soluble proteins could be optimized for large-scale GPCR expression.

Here we report using commercial cell-free translation systems with Brij-35 for the rapid, high-yield production of 13 GPCRs (Table 1), including 9 olfactory receptors (ORs), one human trace-amine receptor (hTAAR5), one human formyl peptide receptor (hFPR3), and 2 human vomeronasal receptors (hVN1R1 and hVN1R5). The expressed GPCRs could be purified to >90%, were properly folded, and were able to bind their ligands. The GPCR hVN1R1 was stably cloned into HEK293 cells. The HEK293 and cell-free expressed hVN1R1 had comparable structures and binding properties.

**Table 1:** Solubility and maximum yields of GPCRs produced using cell-free *in vitro* translation in the presence of Brij-35.

GPCR	% solubility	Yield (mg)*	GPCR	% solubility	Yield (mg)*
Olfr226	86±8	3.7	hOR17-209	88±4	2.5
mOR33-1	85±2	5.9	hOR17-210	91±2	4.5
mOR103-1	90±4	4.5	hFPR3	83±5	5.5
mOR106-13	86±13	2.4‡	hTAAR5	90±1	4.5
mOR174-4	89±2	2	hVN1R1	88±0.1	0.4
mOR174-9	86±3	6	hVN1R5	85±2	1‡
mOR175-1	81±8	2.5			

* Milligrams of receptor that could be produced in a 10 ml cell-free reaction. These yields were calculated from smaller batches of protein purified using immunoaffinity chromatography. Experiments showed that up to 1 mg/ml of protein could be produced, but that up to half could be lost during the purification process. The yields were determined by spectrophotometer readings.

‡ These yields were calculated by comparing the intensities of the receptor samples against a sample with a known concentration.

## Results

### Systematic Detergent Screens

**Figure 1:**
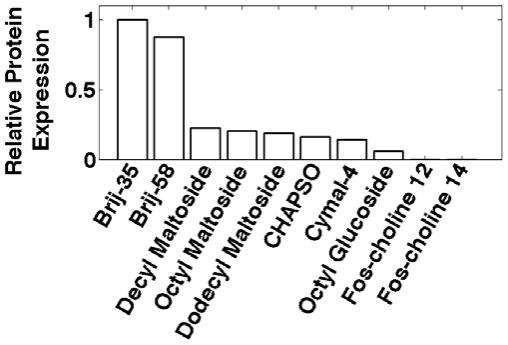
Detergent screen for cell-free GPCR production. Detergents commonly used for membrane protein solubilization or crystallization were screened. Figure 1 shows a detergent screen with the receptor hOR17-210. Brij-35 and Brij-58 yielded ∼4–5 times as much receptor as the next best detergent. Although comparable, Brij-35 consistently had slightly higher yields than Brij-58. Each bar represents the average of 2–3 experiments. The data was normalized to Brij-35.

We systematically screened numerous detergents to assess their ability to produce and solubilize GPCRs expressed in cell-free systems. The chosen detergents included octyl glucoside, decyl maltoside, dodecyl maltoside, CHAPSO (3-[(3-Cholamidopropyl)dimethylammonio]-2-hydroxy-1-propanesulfonate), Brij-35, Brij-58, and fos-choline14 (FC14). These detergents have effectively solubilized and stabilized GPCRs produced in mammalian cells [17], [18], and have been used to obtain high-resolution protein structures. We found few suitable detergents. Brij-35 and Brij-58 yielded ∼4–5 times as much protein as the next best detergent, with Brij-35 consistently achieving slightly higher yields (Figure 1). Our results show that the choice of detergent is critical, and that the detergents commonly used in cell-based production may not be optimal for use in cell-free systems. They may inhibit transcription or translation by interfering with ribosomes or other synthesis machinery.

We then assessed the ability of Brij-35 to solubilize and stabilize multiple GPCRs. Western and dot blot analyses were used to compare soluble and insoluble protein fractions, as well as estimate total protein yields. Without Brij-35, only ∼10% of the produced protein is soluble. With Brij-35, up to ∼93% is soluble (Table 1). Table 1 shows the maximum yield of each GPCR made in Brij-35. These yields are comparable to those obtained from protein expressed in mammalian cells, and are sufficient for biochemical and structural studies. Unlike cell-based protein production, which requires several months, cell-free systems can produce milligrams of protein within hours directly from plasmid DNA. Cell-free production of GPCRs is thus a promising and attractive technique for membrane protein studies.

### Purification of GPCRs from Cell-Free Reactions and HEK293 Cells

We generated a stable inducible HEK293 cell line expressing hVN1R1. The expressed protein was compared to the cell-free counterpart in all subsequent experiments.

**Figure 2:**
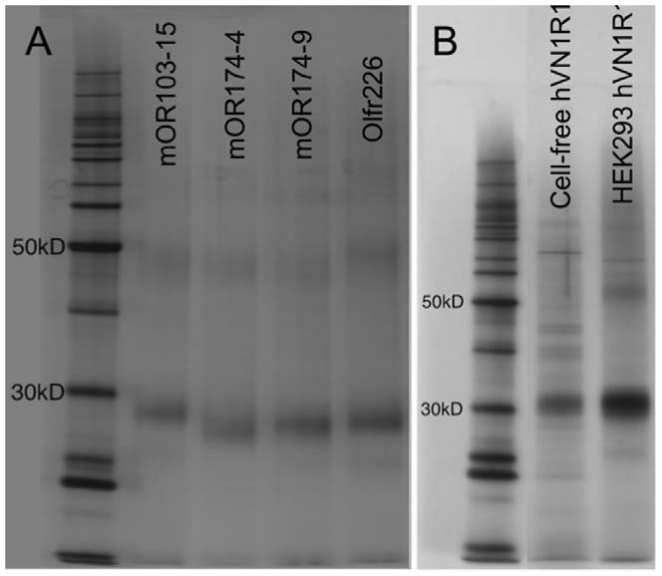
Silver Stains of Purified GPCRs. A) Four cell-free expressed GPCRs. B) Comparison between cell-free and HEK293 expressed hVN1R1. Most GPCRs could be purified to >90% purity, and all showed two bands characteristic of a monomer and a dimer [17], [18]. The cell-free and HEK293 expressed receptors run at the same size, and have similar purities.

We purified several cell-free produced receptors and the HEK293-expressed hVN1R1 using rho-1D4 antibody tagged beads. The purified receptors were up to 90% pure (Figure 2), and could be purified further for crystallization trials using size exclusion chromatography [17], [18]. The receptors were purified in the presence of FC14 because it has been shown to be the optimal detergent for GPCR purification [17]–[20].

### Secondary Structure Analysis Using Circular Dichroism

**Figure 3:**
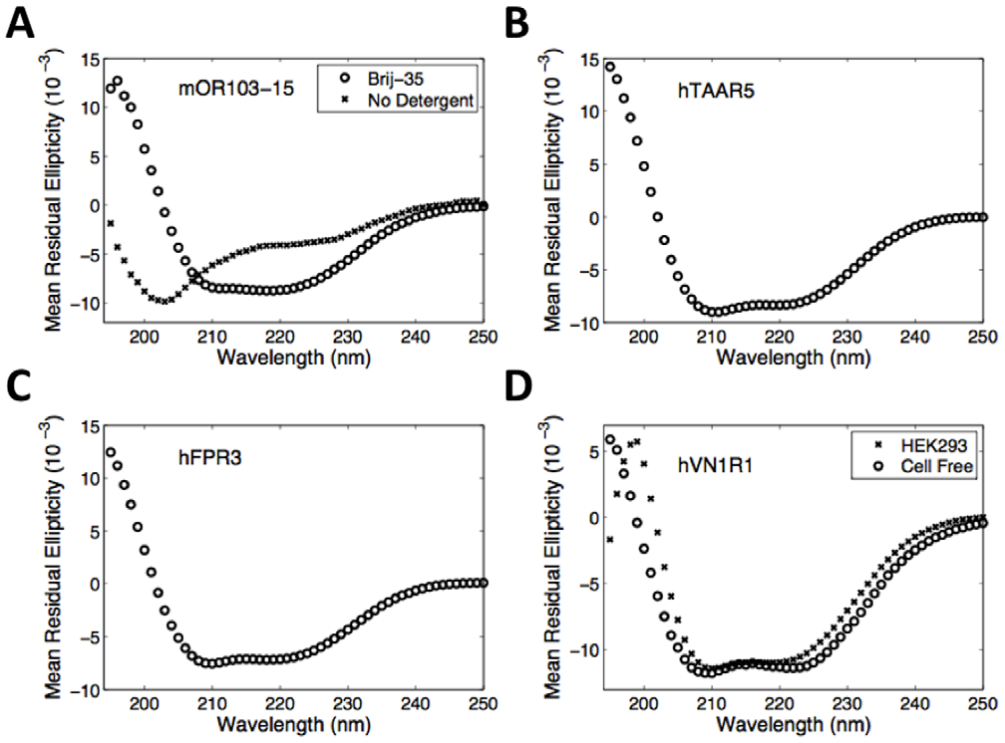
Circular Dichroism Spectra of Five Purified GPCRs. A) Cell-free expressed mOR103-15 made with Brij-35 or no detergent, B) Cell-free expressed hTAAR5, C) Cell-free expressed hFPR3, and D) Cell-free and HEK293 expressed hVN1R1. All purified GPCRs have characteristic alphahelical spectra, except mOR103-15 made without detergent. Since GPCRs have 7-transmembrane helices, and an overall α-helix content of ∼50%, the CD spectra suggest that these receptors are properly folded. The near overlap of the spectra for cell-free and HEK293 expressed hVN1R1 suggests that both receptors are properly folded, and further indicates that cell-free produced GPCRs are comparable to those expressed in mammalian cells.

Circular dichroism (CD) was used to assess the secondary structure of the produced GPCRs. Figure 3 shows the CD spectra of 4 GPCRs purified in detergent, as well as one cell-free control produced without detergent. All of the receptors purified using detergent have characteristic alpha-helical spectra, with signature valleys at 208 nm and 220 nm. The control made without detergent has a spectrum that is more characteristic of a random coil. Because GPCRs have 7-transmembrane α-helical segments, these spectra suggest that the receptors are properly folded and that a detergent is necessary to aid in this process. The near overlap of the spectra for the cell-free and HEK293 hVN1R1 samples indicates that the cell-free reactions produce properly folded receptors. The minor differences in the cell-free and HEK293 curves are likely due to the presence of slight impurities in the samples.

### Ligand-binding Analysis Using Microscale Thermophoresis

Microscale thermophoresis was used to detect binding between the purified GPCRs and their ligands. Microscale thermophoresis is based on the ligand-binding induced change in movement of molecules along a temperature gradient [21], [22]. Unlike surface plasmon resonance (SPR) or other surface-based techniques, thermophoresis is a label-free and surface-free technology that can be used with sample volumes smaller than 5 µl. Thermophoretic molecular gradients are measured in free solution using the fluorescence of a protein's native tryptophans. Immobilization and other coupling chemistries that could alter protein function are thus avoided. Moreover, thermophoresis detects ligands as small as 40Da [23]. Most volatile odorants are less than 300 Da, whereas their receptors are over 30,000 Da. Because of the large mass ratio, these binding interactions are extremely difficult to measure using mass-based technologies, but are possible using microcale thermophoresis [21]–[23].

**Figure 4:**
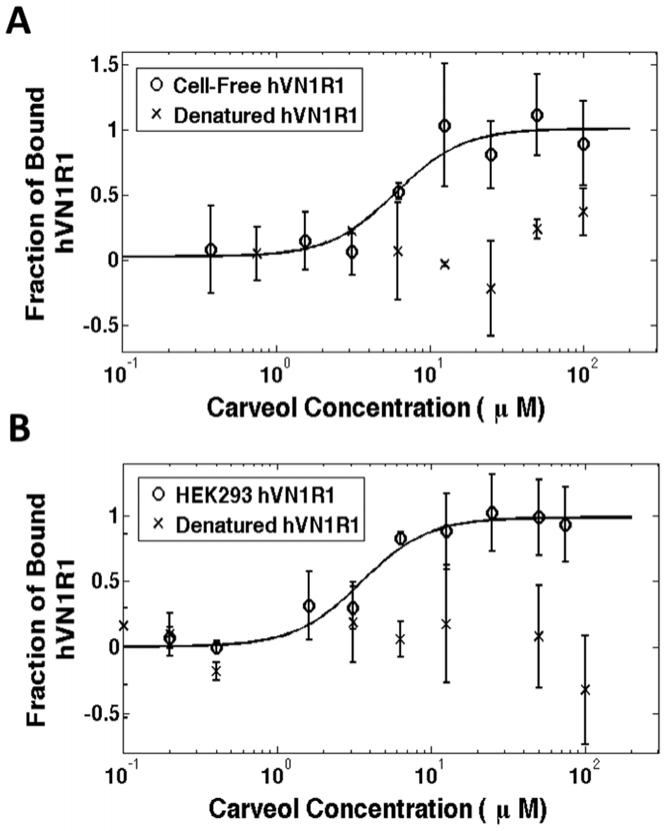
Microscale Thermophoresis Measurements of Purified GPCRs. A) Cell-free expressed hVN1R1 with and without heat-denaturation. B) HEK293 expressed hVN1R1 with and without heat-denaturation. The non-denatured receptors show typical sigmoidal binding curves, with plateaus at low and high concentrations. Cell-free expressed hVN1R1 has an EC_50_ of 6±2 µM, and HEK293 expressed hVN1R1 has an EC_50_ of 3.5±0.7 µM. The heat-denatured controls had flat responses or random amplitudes throughout the ligand titration range. These results show that hVN1R1 is binding carveol. Furthermore, the similar EC_50_ values and binding curves in A) and B) demonstrate that cell-free produced receptors function as well as HEK293 expressed receptors. The curves were normalized to the fraction of bound receptor. Each data point represents the mean of 3 independent experiments; error bars show the standard deviation. The binding curves were fit to the Hill equation. The binding results shown are representative of the data from other binding measurements.

A subset of the receptors used for CD measurements was analyzed for ligand binding. Heat-denatured receptors were used as negative controls. Each native receptor exhibited a typical sigmoidal binding curve, while the heat-denatured controls had random amplitudes throughout the ligand titration range (Figure 4). These results suggest that the cell-free produced GPCRs bound their ligands. All of the measured binding affinities were in the micromolar range, which is consistent with previous reports [14], [17], [24]. The similar binding affinities for cell-free and HEK293 expressed proteins (6±2 µM and 3.5±0.7 µM, respectively) indicate that cell-free produced proteins are functional.

## Discussion

Our study shows that cell-free membrane protein production is a useful technology for expressing milligrams of GPCRs. The receptors could be purified to ∼90% purity using immunoaffinity chromatography alone. CD measurements on a subset of purified GPCRs showed that they had the predicted secondary structures, which suggests that they were properly folded. Microscale thermophoresis indicated that the cell-free produced GPCRs were functional by showing that the purified receptors could bind their reported small-molecule ligands. Comparison of a HEK293 and cell-free expressed protein suggests that cell-free systems are a practical alternative to cell-based platforms for producing GPCRs.

Although cell-free production is a mature technology for soluble proteins, very few membrane proteins have been produced [1]–[16], largely due to the lack of suitable detergents, and laborious detergent screens. In our current study, Brij-35 seemed to consistently be the optimal detergent for olfactory-related GPCRs. Previous reports suggest that, while Brij-35 may not be optimal for all membrane proteins or GPCRs, the Brij family of detergents may function best with cell-free membrane protein expression [3], [14]. While the best detergent for protein production may not be the best detergent for downstream applications, we have shown that a single detergent exchange with FC14 is possible without compromising receptor structure and function. Since FC14 has been used to obtain protein structures [25], [26], it should be possible to couple cell-free expression with crystal screens or NMR structural studies.

In order to accelerate membrane protein structure and function studies, it is absolutely vital to develop simple, straightforward methods of producing sufficient quantities of membrane proteins. Commercial cell free kits offer an attractive alternative to cell-based systems. Milligrams of protein can be produced within hours directly from plasmid DNA. The produced proteins can be purified quickly using conventional methods, and are amenable to detergent exchange for downstream applications. Using commercially available kits, the necessary reagents are easily and widely available, and results are reproducible. Although the 13 GPCRs reported here represent a small fraction of all receptors, it is the largest number presented in a single study with the same methods. Our ability to produce significant quantities of these GPCRs using commercial cell-free systems demonstrates the usefulness of this technology in the field. Indeed, the critical production bottleneck in membrane protein studies may potentially be overcome. Structure and function studies of additional GPCRs may be stimulated and accelerated in the coming years.

## Materials and Methods

### G-Protein Coupled Receptor (GPCR) Gene Design

Protein sequences of 9 olfactory receptors, 2 vomeronasal receptors, one trace amine-associate receptor, and one formyl peptide receptor were obtained from the NCBI online database: hOR17-209 (NP_003546.1), hOR17-210 (SwissProt Q8WZA6.2), mOR31-4 (NP_667290.2), mOR33-1 (GenBank AAL60676.1), mOR103-15 (NP_035113.1), mOR106-13 (NP_001011738.1), mOR171-2 (NP_997547.1), mOR174-4 (GenBank BAB59038.1), mOR174-9 (NP_473431.1), mOR175-1 (SwissProt Q9QY00.1), mOR276-1 (GenBank AAL60877.1), Olfr226 (SwissProt P23270.2), VN1R1 (AF255342), VN1R5 (AY114735), hTAAR5 (NP_003958.2), and hFPR3 (NP_002021.3). The rho1D4 epitope (TETSQVAPA) preceeded by a GSSG linker was added to the C-terminus of each receptor to facilitate purification and western blot detection. The codons for each receptor were optimized for *E. coli* expression. The genes were commercially synthesized by GeneArt (Germany) and subcloned into the pIVex2.3d vector (Roche Diagnostics Corp.) using the NcoI and XhoI restriction sites. The hVN1R1 gene was also subcloned into the pcDNA4T/O vector (Invitrogen) using the EcoRI and XhoI restriction sites. This vector was used for mammalian expression. The final plasmid constructs were verified by DNA sequencing (MIT Biopolymers Labs, Cambridge, MA).

### Cell-free GPCR Production Using Commercial Kits

*E. coli* based cell-free expression kits were used to synthesize the ORs according to the manufacturer's instructions (Invitrogen, K9900-97, Qiagen 32506), with the exception that reactions were performed at 30-33°C. To compensate for the lack of a natural membrane, surfactants were added directly to the reactions. A preliminary screen determined that the optimal concentration was 0.2% w/v. A final reaction volume of 50 µl was used for all screens. After the reactions were complete, the samples were centrifuged at 10,000 rpm for 5 minutes. The supernatant containing the solubilized protein was removed, and the pellet was resuspended in an equivalent volume of PBS. The relative quantities of solubilized and precipitated protein were determined with a western or dot blot. ImageJ (http://rsb.info.nih.gov/ij/) was used to perform all densitometry analyses. Final reaction volumes of 0.5–1.0 ml were used to produce protein that was purified for secondary structure and binding analyses.

### HEK293 GPCR Production

A stable, inducible HEK293 cell line expressing hVN1R1 was generated as previously described [17], [18]. Briefly, plasmid DNA was transfected into HEK293G cells using Lipofectamine 2000 (Invitrogen). The transfected cells were grown in selective media containing Zeocin and Blasticidin until individual resistant colonies were visible. Twenty-four colonies were picked and screened for optimal protein expression. The clone with the highest expression level and least toxicity was selected and amplified for subsequent experiments.

### GPCR Detection and Purity Analysis

Western blots and silver stains were used to detect the proteins and analyze their purity. Samples were prepared and loaded in Novex 10% Bis-Tris SDS-PAGE gels (Invitrogen) according to the manufacturer's protocol, with the exception that the samples were incubated at room temperature prior to loading as boiling causes membrane protein aggregation. For blotting, the gel-resolved samples were transferred to a nitrocellulose membrane, blocked in milk (5% w/v non-fat dried milk in TBST) for 1 hour, and incubated with a rho1D4 primary antibody (1∶3000 in TBST, 1 hour at room temperature, or overnight at 4°C). The GPCRs were then detected with a goat anti-mouse HRP-conjugated secondary antibody (Pierce, Rockford, IL) (1∶5000 in TBST, 1 hour, room temperature) and visualized using the ECL-Plus Kit (GE Healthcare). The SilverXpress kit (Invitrogen, LC6100) was used according to the manufacturer's instructions to perform total protein stains of the samples. All images were captured using a Fluor Chem gel documentaion system (Alpha Innotech, San Leandro, CA). ImageJ software [27], [28] was used to compare band intensities and analyze sample purity.

### Immunoaffinity Purification Using rho1D4 monoclonal antibody

CNBr-activated Sepharose 4B beads (GE Healthcare) chemically linked to the rho1D4 monoclonal antibody (Cell Essentials, Boston, MA) were used for immunoaffinity purification. Solubilized protein from the cell-free reactions was mixed with the bead slurry (binding capacity 0.7 mg/ml) and rotated overnight at 4°C to capture the synthesized protein. The beads were then washed with wash buffer (PBS+0.2% FC-14 w/v) until spectrophotometer readings indicated that all excess protein had been removed (<0.01 mg/ml). The captured GPCRs were eluted with elution buffer (PBS+0.2% FC-14+800 µM elution peptide). The elution peptide Ac-TETSQVAPA-CONH_2_ was synthesized by CPC Scientific Inc., CA. Elutions were performed until spectrophotometer readings indicated that no more protein was present (<0.01 mg/ml). The protein was concentrated using 30 kDa or 50 kDa MWCO filter columns (Millipore, Billerica MA). All concentrations were measured using the NanoDrop 1000 spectrophotometer (Thermo Scientific). For some samples, the concentration was also measured with a total protein stain by comparing the intensity of the GPCR band to the intensity of a BSA band of known concentration. The beads were pelleted by centrifugation at 1,400xg for one minute between each wash and elution.

### Secondary Structural Analysis Using Circular Dichroism

Spectra were recorded on a CD spectrometer (Aviv Biomedical, model 410) at 15°C over the wavelength range of 195–250 nm with a step size of 1 nm and an averaging time of 4 seconds. Spectra for purified GPCRs were blanked to wash buffer. A 111-QS quartz sample cell with a path length of 1 mm (Hellma, USA) was used. 300 µl of protein sample was used for each experiment. The spectra were smoothed using an averaging filter with a span of 5.

### Ligand-Binding Measurements Using Microscale Thermophoresis

Thermophoresis was used to measure the binding interactions between purified receptors and their ligands using a setup similar to that previously described^22^. To eliminate artifacts caused by labeling or modifying proteins, the fluorescence of native GPCR tryptophans was used to monitor the local receptor concentration. For each tested GPCR, a titration series with constant receptor concentration and varying ligand concentrations was prepared in a final solution of 10% DMSO and 0.2% FC-14 in PBS. Potential autofluorescence of each ligand was checked: no fluorescence signal was detected from the ligands in the tryptophan fluorescence channel. The final receptor concentration was 2 µM. Approximately 1.5 µl of each sample was loaded in a fused silica capillary (Polymicro Technologies, Phoenix, USA) with an inner diameter of 300 µm. An infrared laser diode was used to create a 0.12 K/µm temperature gradient inside the capillaries (Furukawa FOL1405-RTV-617-1480, wavelength λ = 1480 nm, 320 mW maximum power, AMS Technologies AG, Münich Germany). The IR-Laser beam couples into the path of fluorescence light with a tailor made UV-transparent hot mirror from AHF-Analysentechnik, and is focused into the fluid with the microscope objective. Tryptophan fluorescence was excited with a UV-LED (285 nm), and was measured with a 40x SUPRASIL synthetic quartz substrate microscope objective, numerical aperture 0.8 (Partec, Goerlitz, Germany). The local receptor concentration in response to the temperature gradient was detected with a photon counter PMT P10PC (Electron Tubes Inc, Rockaway, NJ, USA). All measurements were performed at room temperature. Fluorescence filters for tryptophan (F36-300) were purchased from AHF-Analysentechnik (Tübingen, Germany).
